# Cardiovascular biomarkers: exploring troponin and BNP applications in conditions related to carbon monoxide exposure

**DOI:** 10.1186/s43044-024-00446-w

**Published:** 2024-01-29

**Authors:** Andia Taghdiri

**Affiliations:** Faculty of Medicine, Ivane Javakhishvili Tbilisi State University, Tbilisi, Georgia

**Keywords:** Cardiovascular biomarkers, Troponin, B-type natriuretic peptide (BNP), Carbon monoxide (CO) exposure, Myocardial injury, Cardiovascular complications, High-sensitivity cardiac troponin (hsTnI)

## Abstract

**Background:**

The diagnosis and prognosis of cardiovascular disorders are greatly aided by cardiovascular biomarkers. The uses of troponin and B-type natriuretic peptide in situations involving carbon monoxide exposure are examined in this narrative review. These biomarkers are important because they help predict outcomes in cardiovascular disorders, track the effectiveness of therapy, and influence therapeutic choices.

**Main body:**

Clinical practice makes considerable use of B-type natriuretic peptide (BNP), which has diuretic and vasodilatory effects, and troponin, a particular marker for myocardial injury. Carbon monoxide (CO) poisoning is a major worldwide health problem because CO, a “silent killer,” has significant clinical consequences. Higher risk of cardiac problems, poorer clinical outcomes, and greater severity of carbon monoxide poisoning are all linked to elevated troponin and B-type natriuretic peptide levels. BNP’s adaptability in diagnosing cardiac dysfunction and directing decisions for hyperbaric oxygen therapy is complemented by troponin’s specificity in identifying CO-induced myocardial damage. When combined, they improve the accuracy of carbon monoxide poisoning diagnoses and offer a thorough understanding of cardiac pathophysiology.

**Conclusions:**

To sum up, this review emphasizes the importance of troponin and B-type natriuretic peptide (BNP) as cardiac indicators during carbon monoxide exposure. While BNP predicts long-term cardiac problems, troponin is better at short-term morbidity and death prediction. When highly sensitive troponin I (hsTnI) and B-type natriuretic peptide are combined, the diagnostic accuracy of carbon monoxide poisoning patients is improved. One of the difficulties is evaluating biomarker levels since carbon monoxide poisoning symptoms are not always clear-cut. Accurate diagnosis and treatment depend on the investigation of new biomarkers and the use of standardized diagnostic criteria. The results advance the use of cardiovascular biomarkers in the intricate field of carbon monoxide exposure.

## Background

Cardiovascular illnesses (CVDs), such as coronary artery disease, heart failure, and stroke, are diagnosed, tracked, and prognosed with the use of cardiac biomarkers, which are substances released into the bloodstream by the heart in response to damage or stress [1, 2]. These biomarkers play a critical role in guiding clinical judgments and treatment plans, provide information on the extent, nature, and causes of cardiac injury, and forecasting the probability of subsequent cardiovascular events [3, 4]. Ongoing efforts are centered on creating and verifying novel biomarkers in order to improve our comprehension of the pathophysiology, causes, and subtypes of cardiovascular disease [5].

Among the newly identified biomarkers for CVD are circulating endothelial cells (CECs), soluble ST2 (sST2), microRNAs (miRNAs), galectin-3 (Gal-3), growth differentiation factor-15 (GDF-15), and high-sensitivity C-reactive protein (hs-CRP) [6, 7]. However, there are obstacles and restrictions to the present use of biomarkers, such as problems with cost-effectiveness, accessibility, variability, interference, standardization, and interpretation [5].

Clinical practice uses two widely utilized cardiac biomarkers: troponin and B-type natriuretic peptide (BNP). When heart muscle cells are injured, as in myocardial infarction or myocardial damage, troponin, a protein complex that controls heart muscle cell contraction and relaxation, is released into the bloodstream [8, 9]. Troponin functions as a particular marker for myocardial injury as it is highly expressed in heart tissue and is present in low amounts in healthy people [10, 11]. On the other hand, BNP, a hormone secreted by cardiac muscle cells in reaction to overstretching or elevated pressure, has vasodilatory, natriuretic, and diuretic properties that help lower cardiac strain and preserve fluid and electrolyte balance [12, 13]. BNP levels aid in the diagnosis of heart failure, evaluation of the condition’s severity and origin, tracking of treatment response, prognostic prediction, and risk stratification [14, 15].

Known as a “silent killer,” carbon monoxide (CO) binds to hemoglobin (Hb) to decrease its ability to transport oxygen, which results in CO poisoning (COP) [16]. This odorless, colorless, very poisonous gas can cause tissue damage and severe hypoxia [17]. CO poisoning’s severe clinical symptoms and high morbidity and death rate make it a major worldwide health problem [18]. CO poisoning is more common in those with chronic heart disease, anemia, or respiratory conditions [19, 20]. With 40% of those examined showing increased cardiac biomarkers, myocardial damage is prevalent in CO poisoning cases. This can lead to ischemia, arrhythmias, heart failure, cognitive decline, seizures, and coma [17, 18, 21].

Based on a study conducted in Lanzhou, China, the relative risks of daily ER visits were found to be 1.041 for total cardiovascular disease (CVD), 1.065 for ischemic heart disease (IHD), 1.083 for heart rhythm disturbances (HRD), 1.062 for heart failure (HF), and 1.057 for cerebrovascular diseases (CD) for every 1 mg/m3 increase in CO concentration [22]. Several investigations have examined the function of BNP and troponin in the diagnosis and prognosis of CO poisoning [17, 21, 23]. Greater levels of these biomarkers have been linked to poorer clinical outcomes, a greater risk of cardiac problems, and a worsening of CO poisoning [24, 25].

The primary aim of this comprehensive narrative review is to investigate the applications of troponin and B-type natriuretic peptide (BNP) in conditions associated with carbon monoxide (CO) exposure. This investigation includes a thorough examination of their functions as cardiac biomarkers in the identification, treatment, and outcome of CO poisoning victims. With regard to the use of troponin and BNP in CO-related cardiovascular disorders, the review seeks to give a comprehensive knowledge of the processes, constraints, and future approaches.

## Main body

### Troponin as a cardiac biomarker

In both skeletal and cardiac muscles, the troponin protein complex—which is made up of troponin C (TnC), troponin I (cTnI), and troponin T (cTnT)—controls muscular contraction [26, 27]. Muscle contraction and tropomyosin relocation are caused by a conformational shift in cTnI caused by TnC’s binding to calcium ions [27]. Actin-myosin interaction is inhibited by cTnI, whereas cTnT binds to tropomyosin to secure the troponin complex to the thin filament [27]. In comparison to troponin T, troponin I is eliminated more quickly due to genetic variations, post-translational modifications, and analytical interferences [28, 29].

Low cTnI and cTnT levels can be found using high-sensitivity cardiac troponin (hs-cTn) tests, which helps in CVD diagnosis [30]. Significant unfavorable cardiac and cerebrovascular events (MACCE) are associated with higher hs-cTn levels [30, 31]. Nevertheless, illnesses including renal failure, sepsis, pulmonary embolism, and atrial fibrillation can also result in an increase in hs-cTn [32, 33].

The mechanics of carbon monoxide (CO) poisoning are yet unknown. Proposed processes include CO-induced hypoxia producing cardiomyocyte necrosis [34, 35], oxidative stress activating proteases [36, 37], and apoptosis inducing membrane blebs with cTn release [38–40]. The degree of exposure, length of time, underlying heart condition, and measurement time all affect troponin elevation patterns [17, 21, 41]. Research indicates that peak troponin levels occur 24 h after CO exposure, which helps with both short- and long-term outcome prediction [42]. Increased mortality, neurological aftereffects, and cardiac problems are linked to elevated troponin levels [21].

Troponin helps to diagnose ischemia (type 2 MI) and CO-induced myocardial infarction (type 1 MI), forecast prognosis, and direct treatment [43–45]. In cases of CO poisoning, novel troponin I assays such as those employed by Patel et al. [21] (Table 1) link elevated troponin I to increased mortality, intensive care admission, and intubation. A different research by Koga et al. [17] (Table 1) relates troponin I levels and corrected QT dispersion to successive disability following CO poisoning.

**Table 1:** Troponin I and BNP levels as prognostic biomarkers in carbon monoxide poisoning—summary of key findings

Author	Patel et al. [21]	Koga et al. [17]	Ashry et al. [49]	Turan et al. [50]
Gender	58.8% males, 35.3% non-White	42 men, 28 women	Male: 29 (69%) Female: 13 (31%)	Male gender, younger age
Mean age	51.2	52 ± 18	27.1 ± 12.2	Children aged 0–17 years
Study design	Observational study	Retrospective study	Observational study	Observational study
Study period	1 January 2012 and 31 August 2019	June 2013 and September 2019	December 2016 and May 2017	October 2017 and April 2019
Study population	119 patients with CO poisoning	70 patients with CO poisoning	42 patients with acute CO poisoning	Children with CO poisoning
Troponin Level	Used as a predictor	Used as a predictor	Not mentioned	Not mentioned
BNP Level	Not mentioned	Not mentioned	Used as a predictor	Used as a predictor
Key Findings	22 patients (18.5%) experienced myocardial damage, which was linked to a higher risk of intubation and critical care unit hospitalization	Based on the corrected QT dispersion and the troponin I level, it is possible to estimate the prognosis of patients after CO poisoning	BNP levels are elevated in individuals with acute CO poisoning	A higher NT-proBNP level (> 480 pg/ml) might be a valuable biomarker for the early identification of myocardial damage caused by carbon monoxide
Conclusions	Elevation of TnI was linked to increased mortality inside hospitals	Patients with myocardial injury should have their prognosis and neurological and cardiovascular outcomes examined in addition to their cardiovascular outcomes	A precise, trustworthy biomarker of cardiac damage in patients suffering from acute CO poisoning may be plasma BNP levels	When left ventricular ejection fraction is decreased and myocardial damage brought on by carbon monoxide is present, NT-proBNP may be helpful in detecting it early

CO: Carbon monoxide, BNP: B-Type Natriuretic Peptide, TnI: Troponin I, NT-proBNP: N-Terminal pro-B-Type Natriuretic Peptide, pg/ml: picograms per milliliter

### B-type natriuretic peptide (BNP) as a cardiac biomarker

It is difficult to comprehend the pathophysiology of B-type Natriuretic Peptide (BNP) in carbon monoxide (CO) poisoning, since there are several possible causes that include inflammation, oxidative stress, acidosis, and hypoxia brought on by CO [12, 46–48]. According to a number of research, BNP, which indicates the degree of cardiac damage and dysfunction, is a useful biomarker for both diagnosing and predicting CO poisoning [48, 49]. Moreover, BNP may have a protective effect by reducing fluid retention and vasoconstriction brought on by CO, which enhances cardiac output and tissue perfusion [23, 49, 50].

Reduced left ventricular ejection fraction (LVEF), a crucial indicator of cardiac failure in CO poisoning, may be found with the use of BNP levels [19]. BNP levels are important when choosing hyperbaric oxygen therapy (HBOT), which is the main treatment for CO poisoning. It can lower the risk of heart problems and increase overall survival [19]. BNP levels also show predictive power for the development of delayed neurological sequelae (DNS), which are severe consequences impairing cognitive and motor functions, even though they have a stronger correlation with poor neurologic outcomes in acute CO poisoning [32, 48, 51, 52].

Patients with acute CO poisoning had considerably higher plasma BNP levels than healthy controls in a study by Ashry et al. [49] (Table 1). The degree of CO exposure and the existence of ischemia alterations on electrocardiography (ECG) were linked with these values. The usefulness of NT-proBNP as a precocious biomarker of CO-induced myocardial damage in children was demonstrated by Turan et al. [50] (Table 1), indicating that it may be a perfect biomarker for the early identification of symptoms of CO-induced myocardial injury and decreased left ventricular ejection fraction.

### Comparative assessment

Both biomarkers rise within hours after CO exposure and correlate with the degree of exposure, indicating that troponin and BNP have similar sensitivity in identifying myocardial damage and dysfunction in CO-related situations [23, 52]. However, as BNP may be raised by other conditions such sepsis, pulmonary hypertension, and renal impairment, troponin shows better specificity than BNP in diagnosing cardiac injury brought on by CO exposure [23, 48]. They have different predictive values: BNP is better at predicting long-term cardiac problems and heart failure, whereas troponin is better at predicting short-term mortality and morbidity [52]. In order to provide a thorough picture of cardiac pathology, these biomarkers work in concert to test for myocardial damage and dysfunction in CO poisoning [17, 23, 53]. In CO poisoning, a combination of BNP and highly sensitive troponin I (hsTnI) can improve prognostic value and diagnostic accuracy [17, 23, 53]. Additionally, ECG values have a strong predictive capacity for BNP and troponin I levels, which helps patients with CO poisoning detect and avoid cardiac toxicity early [51].

### Limitations of troponin and BNP in CO-related conditions

The effects of carbon monoxide (CO) exposure on cardiovascular health might be challenging to diagnose since the symptoms are vague, making high-flow oxygen or hyperbaric oxygen therapy necessary right away [18, 54]. Even after early recovery, individuals may experience delayed neurological consequences, making the prognosis potentially unfavorable [18, 54]. Public awareness, education, and the installation of CO detectors in homes and workplaces are necessary components of prevention measures [18, 54].

With regard to CO exposure, troponin and BNP have some limitations, such as limited specificity for CO-induced cardiomyopathy due to their susceptibility to other factors that may cause cardiac damage or dysfunction, such as ischemia, inflammation, infection, or renal failure [23, 48]. Consequently, it is important to evaluate troponin and BNP levels in combination with electrocardiography, echocardiography, and clinical characteristics [23, 48]. Furthermore, the sensitivity of troponin and BNP to CO-induced cardiomyopathy varies, contingent upon exposure intensity, time of testing, and co-occurring conditions [23, 48]. Therefore, it is important to assess troponin and BNP levels as soon as possible following CO exposure; repeated measures may be required to identify variations over time [23, 48]. Additionally, the cut-off values and reference ranges for troponin and BNP vary depending on the assay and population, which affects the predictive and diagnostic efficacy of these markers [23, 48]. Consequently, it is critical to compare the results with the baseline features of the patient and the normal values of the particular test [23, 48].

### Future directions and considerations

Even while troponin and B-type natriuretic peptide (BNP) play important roles in CO-related illnesses, there are still a number of issues that need for more study and advancement. The lack of established and verified reference ranges for troponin and BNP in CO-related circumstances is one of these difficulties, since various techniques, assays, and cut-off values may have an impact on sensitivity and specificity [55, 56]. In order to account for patient variability, efforts should be focused on developing reliable and repeatable reference ranges unique to CO-related disorders [25].

It is essential to comprehend how confounding variables affect troponin and BNP levels in CO-related circumstances. The interpretation of results may be complicated by variables that independently alter these indicators, including hypoxia, inflammation, oxidative stress, renal impairment, and drug usage [48]. Accurate interpretation requires elucidating the routes and processes relating CO exposure to cardiac biomarkers while accounting for relevant confounders [52].

Future study should focus on maximizing the frequency and timing of troponin and BNP measurements in CO-related situations. The degree and duration of CO exposure, the degree and reversibility of heart injury, and the response to therapy are some of the variables that may affect the kinetics and dynamics of these biomarkers [23, 48]. It is crucial to ascertain the ideal time and interval for monitoring these biomarkers in order to record peak and nadir levels and evaluate changes over time [23, 48].

Future research should concentrate on improving diagnostic criteria in order to fill in these research gaps and improve outcomes for individuals with carbon monoxide poisoning and cardiovascular involvement. It is essential to develop and validate defined criteria based on a mix of clinical characteristics, electrocardiography, echocardiography, cardiac magnetic resonance imaging, and biomarkers for the identification of cardiovascular problems associated to CO [57, 58]. Accurate diagnosis can be aided by evaluating the effectiveness of several diagnostic modalities and determining which combination works best in certain situations [58].

Another exciting line of inquiry is the investigation of new biomarkers, such as oxidative stress, inflammation, apoptosis, and mitochondrial dysfunction, that represent the pathophysiology of CO-induced cardiac damage [57, 58]. Evaluating these biomarkers’ predictive significance and how they relate to clinical outcomes will further our knowledge of CO-related cardiovascular disorders [58].

Furthermore, performing randomized controlled trials will yield important insights into the safety and effectiveness of various treatments for CO-related cardiovascular conditions, including antioxidants, anti-inflammatory drugs, hyperbaric oxygen therapy, oxygen therapy, and mechanical circulatory support [57, 58]. Improving outcomes requires the establishment of evidence-based procedures and guidelines for the best care of these patients [58]. All things considered, it should be the goal of future research projects to fill up these gaps and enhance clinical expertise and understanding in the treatment of cardiovascular diseases linked to CO.

## Conclusions

To sum up, this narrative review clarifies the important functions that BNP and troponin play as vital cardiac biomarkers in relation to carbon monoxide (CO) exposure. The thorough investigation of troponin demonstrates its specificity in detecting CO-induced myocardial injury, assisting in the prognosis of immediate cardiac problems. Conversely, BNP shows promise as a multipurpose biomarker that may be used to identify heart failure, inform decisions regarding hyperbaric oxygen therapy, and forecast short- and long-term consequences, such as postponed neurological consequences.

A thorough comprehension of cardiac pathology is provided by the combination of troponin and BNP in the screening process for myocardial damage and dysfunction in cases of CO poisoning. BNP is useful in predicting long-term cardiac problems including heart failure, but troponin is excellent in predicting short-term morbidity and death. Notably, in situations of CO poisoning, the combination of BNP and highly sensitive troponin I (hsTnI) improves prognostic value and diagnostic accuracy.

The difficulties and restrictions that have been mentioned, such variations in sensitivity and specificity, highlight how crucial it is to carefully evaluate troponin and BNP values in relation to CO exposure. In addition, the possible consequences for clinical practice are emphasized, stressing the necessity of developing uniform diagnostic criteria and investigating new biomarkers in order to improve diagnostic precision.

Troponin and BNP levels provide useful information for timely and focused actions in clinical settings, which may enhance patient outcomes. These biomarkers’ diverse functions in CO-related illnesses highlight how important it is to include them in prognostic and diagnostic plans. To improve our knowledge and treatment of CO-related cardiovascular disorders, future research should focus on addressing existing gaps in the field, developing uniform reference ranges, and investigating new biomarkers. All things considered, the information offered in this study adds to the changing picture of cardiovascular biomarker use in the intricate field of CO exposure.

## Data Availability

All data and materials used in this research are freely available in electronic databases of PubMed, Google scholar and Elsevier. References have been provided.

## Abbreviations

CVD: Cardiovascular diseases
CECs: Circulating endothelial cells
sST2: Soluble ST2
miRNAs: MicroRNAs
Gal-3: Galectin-3
GDF-15: Growth differentiation Factor-15
hs-CRP: High-sensitivity C-Reactive protein
BNP: B-Type natriuretic peptide
CO: Carbon monoxide
Hb: Hemoglobin
COP: CO poisoning
IHD: Ischemic heart disease
HRD: Heart rhythm disturbances
HF: Heart failure
CD: Cerebrovascular diseases
TnC: Troponin C
cTnI: Troponin I
cTnT: Troponin T
hs-cTn: High-sensitivity cardiac troponin
MACCE: Major adverse cardiac and cerebrovascular events
MI: Myocardial infarction
LVEF: Left ventricular ejection fraction
HBOT: Hyperbaric oxygen therapy
DNS: Delayed neurological sequelae
ECG: Electrocardiography
NT-proBNP: N-terminal pro-B-type natriuretic peptide
HsTnI: Highly sensitive troponin I
